# Early Prediction of 90-Day Periprosthetic Joint Infection After Hip Arthroplasty for Proximal Femur Fracture Using Machine Learning: Development and Temporal Validation of a Predictive Model

**DOI:** 10.3390/jcm15041668

**Published:** 2026-02-23

**Authors:** Nicolò Giuseppe Biavardi, Francesco Pezone, Federico Morlini, Mattia Alessio-Mazzola, Valerio Pace, Pierluigi Antinolfi, Giacomo Placella, Vincenzo Salini

**Affiliations:** 1 Department of Othopaedics and Traumatology, Vita-Salute San Raffaele University, Via Olgettina 60, 20132 Milan, Italy; 2 Department of Orthopaedics and Traumatology, IRCCS Ospedale San Raffaele, Via Olgettina 60, 20132 Milan, Italy; 3 Trauma & Orthopaedics Department, “MVT-Pantalla” Hospital, USL Umbria 1, Via del Buda, 06059 Pantalla, Italy; 4 Trauma & Orthopaedics Department, “Azienda Osp. S.M. Terni” Hospital, Viale Tristano di Joannuccio, 05100 Terni, Italy

**Keywords:** periprosthetic joint infection, hip fracture, proximal femur fracture, hip arthroplasty, machine learning, XGBoost, risk prediction, EBJIS 2021

## Abstract

**Background:** Periprosthetic joint infection (PJI) after hip arthroplasty for proximal femur fracture is a severe complication, and early postoperative identification remains challenging. This study developed and validated machine learning (ML) models for the early prediction of 90-day EBJIS 2021 “confirmed” PJI using routinely available perioperative data. **Methods:** We performed a single-center retrospective study including 1182 consecutive adults undergoing primary hip arthroplasty for proximal femur fracture (2015–2022). Forty-seven perioperative candidate predictors were extracted, including early postoperative laboratory values (postoperative day 1–2 and maxima within 72 h). Six algorithms were trained and compared (logistic regression, random forest, support vector machine, multilayer perceptron, XGBoost, and stacking ensemble) using a stratified 80/20 training–test split with 10-fold cross-validation, grid-search hyperparameter tuning, and class weighting. A sensitivity-prioritizing classification threshold was derived using training data only and applied unchanged to evaluation cohorts. Uncertainty was estimated via 1000 bootstrap iterations. Calibration was assessed using the Brier score and calibration intercept/slope. Temporal validation was conducted in a same-center 2023 cohort (*n* = 147). Model explainability used SHAP. **Results:** EBJIS-confirmed 90-day PJI occurred in 58/1182 (4.9%) patients. In held-out testing, the final XGBoost model demonstrated good discrimination (AUC 0.889, 95% CI 0.804–0.960) with good overall calibration (Brier score 0.043). Using a prespecified sensitivity-prioritizing threshold selected in the training set, test-set sensitivity was 100%, specificity 58.5%, PPV 11.4%, and NPV 100%. The stacking ensemble yielded the highest discrimination (AUC 0.937; 95% CI 0.89–0.98). In temporal validation (same-center 2023 cohort; *n* = 147), model performance remained stable (AUC 0.892; sensitivity 85.7%; NPV 99.1% at the prespecified threshold). Calibration was favorable in the development cohort (Brier 0.041; intercept −0.04; slope 0.96) and in 2023 (Brier 0.038; intercept −0.06; slope 0.94). SHAP identified postoperative C-reactive protein, operative duration, body mass index, ASA class, and serum sodium as the most influential predictors. **Conclusions:** ML models, particularly XGBoost, supported early postoperative risk stratification for 90-day EBJIS-confirmed PJI after fracture-related hip arthroplasty, with a consistently high NPV and stable calibration in a temporally independent same-center cohort. Prospective multi-center validation and impact evaluation are needed before clinical implementation.

## 1. Introduction

Periprosthetic joint infection (PJI) remains one of the most severe complications after hip arthroplasty, frequently requiring prolonged antimicrobial therapy and complex reoperations (e.g., DAIR, one- or two-stage revision) and imposing a major health–economic burden on healthcare systems [1,2]. This concern is amplified when arthroplasty is performed for proximal femur (hip) fractures, where surgery is typically unplanned/urgent and delivered to frail, comorbid older patients who cannot be optimized preoperatively to the same degree as elective candidates [3,4]. In this population, reported infection rates vary substantially depending on definitions and follow-up: a recent systematic review/meta-analysis of hemiarthroplasty for femoral neck fractures found pooled rates of ~1.3% superficial SSI and ~2.1% deep SSI, with higher rates in studies using defined diagnostic criteria (deep SSI pooled ~2.9%, upper CI approaching ~4.9%) [5]. Large contemporary cohort studies similarly report PJI/SSI rates around ~1.7–2.3%, underscoring both clinical relevance and heterogeneity in case-mix and surveillance intensity [3,4].

The timely identification of PJI remains clinically challenging, particularly in the immediate postoperative period. Physiological postoperative inflammation can confound clinical assessment, and commonly used serum and synovial biomarkers may require different (often higher) diagnostic thresholds than those used for chronic infection [6,7]. Diagnostic uncertainty is further amplified in paucisymptomatic and low-virulence infections, in which CRP/ESR can be normal or only mildly elevated and microbiological confirmation may be more difficult [8,9]. In fracture-related arthroplasty, these challenges are often compounded by high baseline inflammatory activity and a greater burden of comorbidity, which can obscure early signals of infection and complicate decision-making during the index admission.

Contemporary diagnostic frameworks, including the 2018 ICM (Philadelphia) evidence-based criteria and the EBJIS 2021 “traffic-light” definition, have improved standardization [10,11]. Nevertheless, a non-trivial proportion of patients may remain “inconclusive/uncertain” depending on which definition is applied; in a head-to-head comparison, the ICM 2018 criteria produced more inconclusive diagnoses than EBJIS 2021 [12]. Because delayed recognition can push management away from time-sensitive, implant-retaining strategies and toward more complex revision pathways, there is a need for approaches that support early risk stratification soon after surgery [13,14]. Importantly, the clinical decision need in this setting is typically not definitive diagnosis on postoperative day 1, but the prioritization of surveillance and targeted diagnostic assessment during the index admission and at discharge—especially in a population with low event prevalence where indiscriminate testing can increase unnecessary investigations and workload.

Artificial intelligence (AI) methods, and in particular machine learning (ML), offer a complementary approach to conventional risk assessment by integrating multiple perioperative variables and modeling non-linear interactions that are difficult to synthesize reliably at the bedside [15,16]. In the recent literature on PJI/SSI prediction and detection around arthroplasty, ML-enabled tools have shown promising discrimination, including reports of very high AUCs in selected settings (e.g., automated SSI surveillance and diagnostic modeling) [17,18]. These systems have been proposed as decision-support tools to improve surveillance efficiency while maintaining a high negative predictive value, potentially reducing manual review workload in low-risk cases [17,19]. However, there remains a practical need for models specifically developed for the high-risk post-fracture arthroplasty population, with a clear reporting of the intended timing and use case, transparent methodology, and validation in an independent cohort—standards increasingly emphasized by TRIPOD + AI and PROBAST + AI [20,21].

The objective of the present study was, therefore, to develop and validate ML-based predictive models for the early postoperative risk stratification of PJI within 90 days after hip arthroplasty performed for proximal femur fracture. To maximize label specificity for model development, PJI status was adjudicated using the EBJIS 2021 definition and “confirmed” PJI was used as the positive outcome [11]. We compared the predictive performance of multiple algorithms and explored model explainability to identify perioperative features most strongly associated with PJI risk. Our overarching aim was to support clinically actionable stratification using data available in the early postoperative window (approximately the first 48–72 h), as a decision-support aid to prioritize surveillance and diagnostic assessment rather than a substitute for established diagnostic criteria.

## 2. Materials and Methods

### 2.1. Study Design and Setting

We conducted a single-center, retrospective observational study at IRCCS San Raffaele. At the time of surgery, all patients provided written informed consent for the use of their clinical data for research purposes, in accordance with institutional policy. All data were pseudonymized prior to analysis.

### 2.2. Participants and Study Period

All consecutive adults (≥18 years) who underwent primary hip arthroplasty for proximal femur fracture between January 2015 and December 2022 were eligible for the development cohort. The study period was selected to ensure consistent availability of structured electronic health records and laboratory data for all candidate predictors across the entire interval. Only primary implants were included; revision procedures were excluded because baseline risk, surgical complexity, and diagnostic pathways differ substantially from primary fracture arthroplasty. Exclusion criteria were documented pre-existing infection, severe immunodepression, polytrauma, pathological fractures, and prior ipsilateral surgery.

Inclusion required documentation sufficient to determine infection status through postoperative day 90 (e.g., outpatient records, readmissions, microbiology, or documented clinical contact). Patients without adequate information to adjudicate the 90-day endpoint were not included. A temporally independent same-center cohort including consecutive eligible patients treated in calendar year 2023 was used for temporal validation.

### 2.3. Outcome Definition and Adjudication

The primary outcome was periprosthetic joint infection within 90 days, defined using the EBJIS 2021 criteria. For binary model development, only EBJIS “confirmed” infections were labeled as outcome-positive to maximize label specificity. Suspected cases were adjudicated independently by two reviewers (one orthopedic surgeon and one infectious diseases specialist) based on clinical documentation and the available diagnostic work-up. Diagnostic work-up included joint aspiration when clinically indicated and microbiological assessment from intraoperative sampling. When surgery was performed for suspected infection, at least three intraoperative culture specimens were collected.

### 2.4. Candidate Predictors and Prediction Window

Clinical data were extracted from institutional electronic health records (including operative and anesthesia reports) and the laboratory information system. Forty-seven candidate predictors were prespecified and grouped a priori into: (i) demographic/anthropometric characteristics (age, sex, BMI); (ii) baseline health status (comorbidities and ASA classification); (iii) fracture-related variables (fracture type); (iv) intraoperative variables (including operative duration and surgical technique); (v) implant-related variables (including cementation); and (vi) perioperative laboratory parameters (including CRP, ESR, leukocyte count, and serum sodium). In contrast to some prior approaches, length of stay and early postoperative complications were not included as model predictors to minimize the risk of incorporating downstream care process variables.

Predictors were restricted to information available during the perioperative period and early postoperative course, before outcome determination. Preoperative laboratory values were defined as measurements obtained within 24 h before surgery. Postoperative laboratory data included values from postoperative day (POD) 1 and POD 2; when multiple measurements were available, we retained the maximum value within the first 72 postoperative hours. Perioperative transfusion status was defined as occurring during the index admission and recorded from routine clinical documentation. The model was designed for early in-hospital/discharge risk stratification (approximately 48–72 h from surgery) to prioritize surveillance and targeted diagnostic assessment rather than to replace established diagnostic criteria.

### 2.5. Risk of Reverse Causality and Information Leakage

Because the model is intended for early postoperative risk stratification, some predictors—particularly postoperative laboratory values (POD1–2 and maxima within 72 h)—may reflect early evolving physiological responses rather than baseline risk alone. To mitigate information leakage, we restricted predictors to routinely collected perioperative variables available before outcome determination and implemented all preprocessing and model selection strictly within the training data. To quantify the contribution of early postoperative signals, we performed prespecified sensitivity analyses using restricted predictor sets (preoperative/intraoperative only, and POD1-only), comparing discrimination to the full model.

### 2.6. Data Preprocessing and Missing Data

All variables were harmonized to ensure consistent units, definitions, and coding across the study period. Continuous variables were retained on their native scale; for algorithms requiring scaling, z-score standardization was applied using parameters derived exclusively from the training set to prevent information leakage. Categorical variables were encoded using one-hot encoding. Missingness was assessed across all candidate predictors; patients with missing values in any candidate predictor were excluded prior to model development, resulting in a complete-case dataset for the final analysis, and therefore no imputation was performed. No outlier-specific handling procedures were applied.

### 2.7. Model Development and Internal Validation

Six supervised machine learning algorithms were developed and compared: logistic regression, random forest, support vector machine, XGBoost, multilayer perceptron, and a stacking ensemble. The development cohort (2015–2022) was partitioned using a stratified 80/20 training–test split to preserve outcome prevalence. All model development steps, including preprocessing, class-imbalance handling, and hyperparameter optimization, were performed exclusively using data within the training set.

Internal validation and model selection were conducted using 10-fold cross-validation within the training partition. Hyperparameters were optimized via grid search nested within cross-validation. To mitigate the impact of outcome imbalance, class weighting was applied during model training (within each cross-validation fold) to reduce bias toward the majority class while avoiding information leakage. Following model selection, the final model was refit on the full training set and evaluated once on the held-out test set, which remained untouched during development.

### 2.8. Temporal Validation

Temporal validation was performed in a consecutive same-center cohort treated in calendar year 2023 (*n* = 147), using the final model trained on the 2015–2022 development cohort and applying the prespecified decision threshold derived from the development cohort.

### 2.9. Performance Assessment, Uncertainty, Calibration, and Clinical Utility

Discrimination was quantified using the area under the receiver operating characteristic curve (AUC). Classification performance at a single operating point was summarized using sensitivity, specificity, positive predictive value (PPV), and negative predictive value (NPV). The classification threshold was selected in the training set using out-of-fold predictions to achieve sensitivity ≥0.90, maximizing specificity among thresholds meeting this constraint, and was then applied unchanged to the held-out test and temporal validation cohorts.

Uncertainty was assessed using bootstrap resampling (1000 iterations), reporting confidence intervals for the AUC and for operating-point metrics. Calibration was evaluated using calibration plots and calibration intercept/slope; overall calibration error was summarized using the Brier score. Decision curve analysis was performed to estimate the potential net benefit across clinically relevant threshold probabilities.

### 2.10. Model Interpretability

Model interpretability was assessed using SHapley Additive exPlanations (SHAP) to quantify feature contributions at individual and cohort levels. For XGBoost, SHAP values were computed using TreeSHAP; analogous SHAP-based approaches were applied to the remaining models. Explainability analyses were performed on the held-out test set.

### 2.11. Descriptive Comparisons and Sensitivity Analyses

Baseline characteristics were summarized descriptively. Group comparisons between patients with and without PJI used the Mann–Whitney U test for continuous variables and χ^2^ or Fisher’s exact test for categorical variables (two-sided α = 0.05). *p*-values were provided for descriptive purposes and were not used for feature selection, which was handled within the ML pipeline.

Prespecified sensitivity analyses focused on prediction timing and early postoperative information. Specifically, we evaluated restricted feature sets to quantify the contribution of early postoperative laboratory trajectories to discrimination, including (i) a model restricted to preoperative and intraoperative variables only (excluding POD laboratory values) and (ii) a POD1-only model (excluding POD2 variables). All sensitivity models were trained and evaluated using the same preprocessing pipeline, train/test split, and class-imbalance handling as the primary XGBoost analysis to ensure comparability.

### 2.12. Software and Reproducibility

All analyses were performed in Python v. 3.12 using scikit-learn for model development and evaluation, XGBoost for gradient-boosted models, and SHAP for explainability. Reproducibility was supported through fixed random seeds and the implementation of preprocessing and model training within pipeline-based workflows to minimize leakage between development and evaluation. The code and full modeling pipeline can be shared upon reasonable request.

## 3. Results

### 3.1. Study Population and Endpoint Frequency

**Figure 1:**
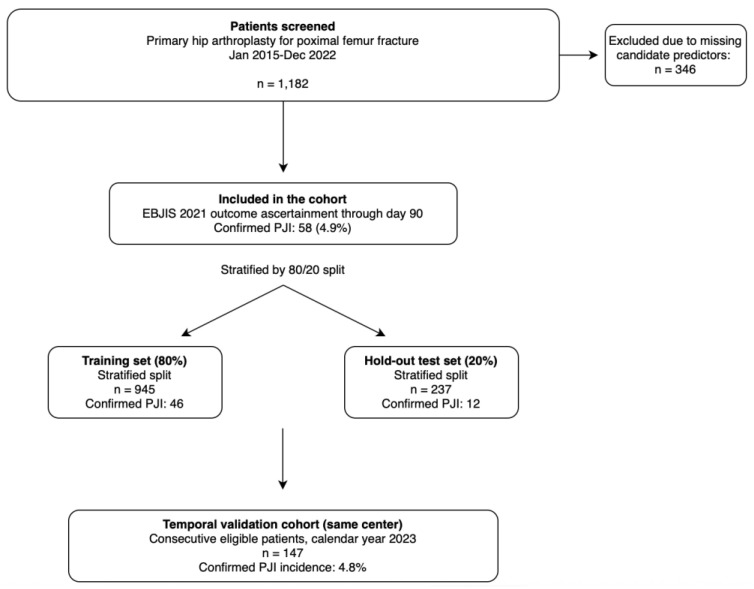
Study flow diagram.

From January 2015 to December 2022, 1182 consecutive adult patients undergoing primary hip arthroplasty for proximal femur fracture met the inclusion criteria for model development. Within 90 days of the index procedure, 58 patients (4.9%) met the EBJIS 2021 “confirmed” definition of PJI and were classified as outcome-positive. Median follow-up in the development cohort was 410 days (IQR 180–640). No patients were excluded due to loss to follow-up before postoperative day 90, consistent with the prespecified endpoint ascertainment requirement. Patient selection and cohort partitioning are summarized in Figure 1.

### 3.2. Patient and Perioperative Characteristics

Baseline demographic, clinical, and perioperative characteristics are summarized in Table 1. The cohort had a median age of 84 years (IQR 77–89), 71% were women, and median BMI was 25.1 kg/m^2^ (IQR 22.8–28.4). Hemiarthroplasty accounted for 82% of procedures and total hip arthroplasty for 18%. Median operative duration was 87.5 min (IQR 60–115), cemented fixation was used in 74%, and perioperative transfusion occurred in 29%. In descriptive comparisons, patients who developed confirmed PJI had longer operative time, and perioperative transfusion was more frequent in the PJI group.

### 3.3. Model Development and Internal Evaluation

Models were developed using a stratified 80/20 training–test split with 10-fold stratified cross-validation within the training partition. Confirmed PJI events were 46 in the training set and 12 in the held-out test set, consistent with stratification. Missingness was assessed across all candidate predictors; patients with missing values in any candidate predictor were excluded prior to model development, resulting in a complete-case dataset for model training and evaluation.

### 3.4. Comparative Model Discrimination and Classification Performance

Across evaluated algorithms, XGBoost provided the strongest performance among single models at the prespecified sensitivity-prioritizing operating point. In held-out testing (*n* = 236; 12 confirmed PJIs), the final XGBoost model achieved an AUC of 0.889 (95% CI 0.804–0.960) and a Brier score of 0.043. Using a sensitivity-prioritizing threshold prespecified from the training set using out-of-fold predictions, the model yielded 100% sensitivity, 58.5% specificity, 11.4% PPV, and a 100% NPV in the test set (TP = 12, FP = 93, TN = 131, FN = 0).

The performance of the remaining models is summarized in Table 2, including logistic regression (AUC 0.842), random forest (AUC 0.881), support vector machine (AUC 0.864), multilayer perceptron (AUC 0.872), and the stacking ensemble (AUC 0.937). Although the stacking ensemble yielded the highest discrimination (AUC 0.937, 95% CI 0.89–0.98), XGBoost was retained as the primary candidate for clinical translation, given its high performance among single models and greater interpretability/implementation simplicity.

### 3.5. Temporal Validation (Same-Center 2023 Cohort)

Model transportability was evaluated in a temporally independent same-center cohort treated in 2023 (*n* = 147), in which the 90-day EBJIS-confirmed PJI incidence was 4.8%. Applying the prespecified threshold derived from the development cohort, XGBoost achieved an AUC of 0.892 (95% CI 0.73–1.00), with sensitivity 85.7%, specificity 82.1%, PPV 19.4%, and NPV 99.1% (Table 3).

### 3.6. Calibration and Risk Stratification Performance

**Figure 2:**
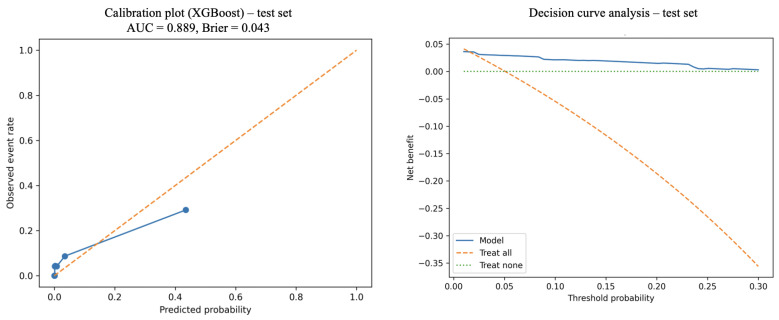
Calibration and decision curve analysis of the XGBoost model in the held-out test set.

In the held-out test set, calibration is shown in Figure 2, and overall calibration error was low (Brier score 0.043). For completeness, calibration in the overall development cohort was summarized by a Brier score of 0.041 with calibration intercept −0.04 and slope 0.96. In the temporally independent 2023 cohort, calibration remained stable (Brier score 0.038, intercept −0.06, slope 0.94). Decision curve analysis demonstrated a net benefit across clinically relevant threshold probabilities in the held-out test set (Figure 2).

### 3.7. Explainability Analysis and Clinically Salient Predictors

**Figure 3:**
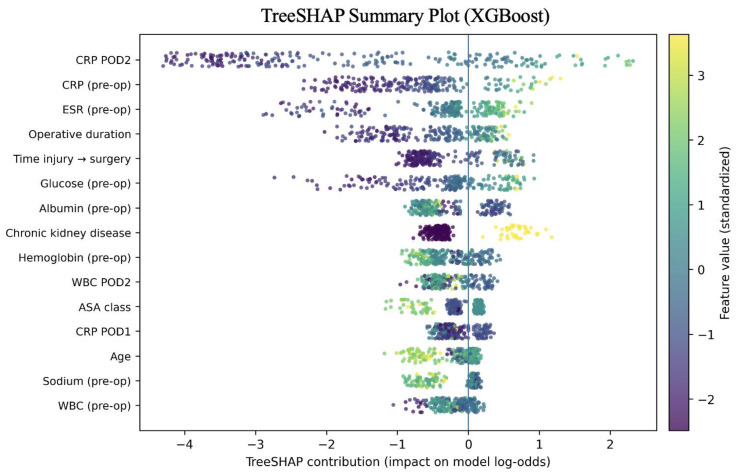
TreeSHAP summary plot of key predictors (XGBoost).

TreeSHAP analyses indicated that a limited subset of perioperative variables accounted for a substantial proportion of risk attribution. Higher postoperative CRP (≈21 mg/L), longer operative duration, higher BMI, higher ASA class, and lower serum sodium were the most influential predictors. These findings are consistent with the intended use case of early postoperative risk stratification within approximately 48–72 h from surgery, reflecting the timing of postoperative laboratory predictors (Figure 3).

### 3.8. Sensitivity Analyses

To assess the dependence of model performance on early postoperative laboratory trajectories, we evaluated restricted feature sets. In the held-out test set, the full model (preoperative, intraoperative, and POD1–2 laboratories) achieved AUC 0.889. When restricted to preoperative and intraoperative variables only (excluding POD laboratory values), discrimination decreased (AUC 0.713). A POD1-only model (excluding POD2 variables) retained intermediate performance (AUC 0.823). These analyses suggest that early postoperative laboratory signals contribute substantially to discrimination, consistent with the model’s intended use for in-hospital/discharge risk stratification rather than purely preoperative risk estimation. All sensitivity models were trained and evaluated using the same preprocessing pipeline, train/test split, and class-imbalance handling as the primary XGBoost analysis to ensure comparability.

## 4. Discussion

This study aimed to develop and validate ML models for the early identification of 90-day PJI after hip arthroplasty performed for proximal femur fracture, using the EBJIS 2021 definition as the reference standard and labeling only “confirmed” infections as outcome-positive [11]. Among 1182 patients in the development cohort (2015–2022), the incidence of EBJIS-confirmed PJI was 4.9%. In held-out test evaluation, XGBoost showed good discrimination in held-out testing (AUC 0.889, 95% CI 0.804–0.960) with a high NPV at a sensitivity-prioritizing operating point, supporting its role as a rule-out-oriented surveillance/triage tool. Temporal evaluation in a same-center 2023 cohort (*n* = 147) demonstrated maintained discrimination and rule-out performance at the prespecified threshold, supporting temporal robustness within the same institution. Calibration was good in both cohorts (Brier 0.041 and 0.038, with slopes close to 1), and decision curve analysis suggested a potential clinical net benefit across relevant thresholds. Collectively, these findings support the feasibility of actionable early postoperative risk stratification in a high-risk fracture arthroplasty population using routinely available perioperative data.

The predictors contributing most strongly to model output include postoperative C-reactive protein (CRP), operative duration, body mass index (BMI), ASA class, and serum sodium. These are clinically coherent and align with established concepts in arthroplasty infection risk: host vulnerability and physiological reserve (e.g., obesity and higher ASA class), procedural complexity and tissue stress (operative duration), and early postoperative inflammatory trajectories (CRP). Notably, the model was explicitly framed around information available within ~48–72 h after surgery, consistent with our predictor timing (POD1–2 laboratories and maxima within 72 h). In sensitivity analyses restricting predictors to preoperative and intraoperative variables only, discrimination decreased substantially, whereas a POD1-only model retained intermediate performance, supporting deployment as an early postoperative surveillance tool rather than a purely preoperative risk calculator.

Our findings are consistent with a growing body of literature applying ML to PJI prediction or detection, most commonly in elective arthroplasty cohorts or registry/administrative datasets. In a national database study of primary THA, Salimy et al. (2025) reported good discrimination for ML-based PJI prediction and highlighted predictors overlapping conceptually with ours, including higher ASA class and lower preoperative sodium [22]. Similarly, Dragosloveanu et al. (2025) evaluated multiple supervised ML models for PJI prediction and found strong performance for tree-based approaches, reinforcing the frequent competitiveness of gradient-boosted methods for tabular clinical data [23]. Importantly, some recent work has moved toward “in-time” ML approaches designed for earlier identification using evolving perioperative signals, conceptually closer to the present study’s early postoperative orientation [24].

Comparability across studies remains constrained by heterogeneity in outcome definitions and labeling conventions. We used EBJIS 2021 and restricted positives to “confirmed” infections, a strategy that increases label specificity but may reduce applicability to the broader clinical spectrum in which “likely”/probable cases can influence decision-making [11,12]. Differences across diagnostic frameworks can materially affect apparent model performance and the proportion of “uncertain” cases [12]. Accordingly, standardized definitions and transparent endpoint adjudication are essential when interpreting ML performance and transportability.

From a clinical implementation perspective, the model’s very high NPV under a sensitivity-prioritizing threshold is particularly relevant for rule-out-oriented triage: patients predicted as low-risk may require less intensive surveillance, while patients flagged as high-risk could undergo structured evaluation. However, the PPV is necessarily constrained by the low event prevalence and the operating point chosen to minimize missed infections; therefore, a “high-risk” prediction should be interpreted as a prompt for targeted work-up rather than diagnostic confirmation. Translating such models into practice also requires attention to usability and interpretability, not just discrimination: in this regard, Du et al. developed a visualized, web-based ML risk prediction system for sarcopenia using cohort data and coupled model output with SHAP-based explanations to support clinician understanding and actionable decision support [25]. A similar implementation paradigm—probability output combined with interpretable feature contributions in a clinician-facing interface—could facilitate adoption of early PJI risk stratification tools and mitigate “black-box” concerns. These considerations, together with the emphasis on calibration, prespecified thresholding, and a clear description of preprocessing and validation procedures, are aligned with contemporary expectations for the transparent reporting and appraisal of AI prediction models (TRIPOD + AI; PROBAST + AI) [21,26].

This study has several strengths. It focuses on a clinically distinct, high-risk population that is underrepresented in many elective arthroplasty prediction cohorts, and it uses a contemporary reference standard (EBJIS 2021) with dual independent adjudication involving orthopedics and infectious disease specialists. Methodological rigor included stratified splitting, cross-validated hyperparameter tuning with class weighting, bootstrap-based confidence intervals, calibration assessment (intercept/slope and Brier score), decision curve analysis, and interpretable modeling using SHAP within pipeline workflows designed to reduce leakage. The addition of a temporally independent 2023 cohort strengthens the evidence for stability over time within the same center environment.

Several limitations should be acknowledged. First, the retrospective single-center design limits generalizability, and temporal validation within the same institution should not be interpreted as multi-center external validation. Second, the number of events (58 confirmed PJIs) is modest relative to the predictor set; despite cross-validation, class weighting, and sensitivity analyses, small-event settings can still be vulnerable to optimism and the instability of effect attribution. Third, restricting positives to EBJIS-confirmed infection may underrepresent clinically meaningful “likely” infections and may affect downstream utility depending on local diagnostic pathways. Finally, a reliance on predictors that mature during the early postoperative course, particularly POD1–2 laboratory values and maxima within 72 h, indicates the model is best used for early postoperative/discharge stratification rather than purely preoperative prediction; this is supported by sensitivity analyses showing reduced discrimination when restricting predictors to preoperative and intraoperative variables only.

Future work should prioritize multi-center external validation, the assessment of model performance under site-to-site variation in laboratory ordering and clinical pathways, and prospective impact studies focused on whether ML-guided prioritization improves the timeliness of diagnostic work-up without increasing missed infections. Consistent with TRIPOD + AI and PROBAST + AI recommendations [21,25], these studies should report calibration, thresholding strategy, and clinically meaningful decision-analytic performance to support safe translation.

## 5. Conclusions

In this single-center cohort of patients undergoing hip arthroplasty for proximal femur fracture, ML models, most notably XGBoost, provided accurate early prediction of 90-day EBJIS-confirmed periprosthetic joint infection. Performance was robust, with a consistently high negative predictive value and good calibration, including in a temporally independent 2023 validation cohort. The main predictors (early postoperative CRP, operative time, BMI, ASA class, and serum sodium) were clinically plausible, supporting the model’s potential as a decision support tool to help prioritize early diagnostic work-up and targeted postoperative surveillance. Prospective, multi-center validation and an evaluation of real-world workflow impact are needed before clinical implementation.

## Figures and Tables

**Table 1:** Baseline demographic, clinical, and perioperative characteristics.

Characteristic	Overall (n = 1182)	PJI (n = 58)	No PJI (n = 1124)	*p*-Value
Age, years median (IQR)	84 (77–89)	83 (78–88)	84 (77–89)	0.22
Female sex *n* (%)	839 (71.0)	41 (70.7)	798 (71)	0.97
BMI, kg/m^2^ median (IQR)	25.1 (22.8–28.4)	26.2 (23.9–29.8)	25.0 (22.7–28.3)	0.12
Procedure type				
Hemiarthroplasty—*n* (%)	969 (82.0)	45 (77.6)	924 (82.2)	0.34
Total hip arthroplasty—*n* (%)	213 (18.0)	13 (22.4)	200 (17.8)	0.34
Operative duration, min median (IQR)	87.5 (60–115)	95 (75–115)	76.5 (60–93)	<0.001
Cemented fixation *n* (%)	875 (74.0)	46 (79.3)	829 (73.8)	0.36
Perioperative transfusion *n* (%)	343 (29.0)	24 (41.4)	319 (28.4)	0.03
Follow-up, days median (IQR)	410 (180–640)	400 (180–620)	411 (182–640)	0.61

**Table 2:** Discrimination and classification performance of candidate models in the held-out test set (20% split).

Model	AUC	95% CI (AUC)	Sensitivity (%)	Specificity (%)	PPV (%)	NPV (%)
Logistic regression	0.842	0.77–0.91	78.6	82.3	18.9	97.1
Random forest	0.881	0.81–0.94	85.7	84.1	22.4	97.8
Support vector machine	0.864	0.79–0.93	82.1	83.5	21.0	97.4
Multilayer perceptron	0.872	0.80–0.94	83.6	84.0	22.0	97.6
XGBoost	0.889	0.80–0.96	100	58.5	11.4	100
Stacking ensemble	0.937	0.89–0.98	92.9	86.9	28.4	98.4

**Table 3:** Temporal validation (2023 same-center cohort): discrimination and classification performance.

Model	AUC	95% CI (AUC)	Sensitivity (%)	Specificity (%)	PPV (%)	NPV (%)
Logistic regression	0.804	0.61–1.00	71.4	80.7	15.6	98.3
Random forest	0.858	0.68–1.00	85.7	82.9	20.0	99.1
Support vector machine	0.835	0.65–1.00	71.4	83.6	17.9	98.3
Multilayer perceptron	0.848	0.67–1.00	85.7	83.6	20.7	99.2
XGBoost	0.892	0.73–1.00	85.7	82.1	19.4	99.1
Stacking ensemble	0.905	0.75–1.00	85.7	85.7	23.1	99.2

## Data Availability

The datasets generated and/or analyzed during the current study are available from the corresponding author upon reasonable request, subject to evaluation by the authors and in accordance with applicable institutional policies and data protection regulations.
